# Late Hemorrhagic Complication and Unilateral Neck Edema Related to Cannulation in Veno-Venous Extracorporeal Membrane Oxygenation (VV ECMO): A Case Report

**DOI:** 10.7759/cureus.79016

**Published:** 2025-02-14

**Authors:** Stanislav Shkolnyi, Viktoriia Matviichuk, Oleksandra Kaliuzhna, Andrii Vysotskyi

**Affiliations:** 1 Department of Pediatric Anesthesiology and Intensive Care Medicine With Extracorporeal Membrane Oxygenation, National Specialized Children’s Hospital Ministry of Health of Ukraine "OHMATDYT", Kyiv, UKR

**Keywords:** cannulation complication, case report, ecmo complication, hemorrhage, neck edema, neck swelling, vv ecmo

## Abstract

We report a case of a late cannulation-related complication on veno-venous extracorporeal membrane oxygenation (VV ECMO), which includes unilateral neck swelling. A 20-year-old woman, post bone marrow transplantation, was admitted to the ICU with respiratory failure. On day 3, due to conventional support failure, VV ECMO was initiated. On day 2 of the ECMO run, significant right-sided neck swelling occurred, with a marked tracheal deviation to the left, which was accompanied by intensive hemoptysis. On day 5 of ECMO, the patient was successfully weaned off ECMO, decannulated without complications, and subsequently extubated. ​​​​​​​A multidisciplinary review of the case concluded that the complication was caused by the partial obstruction of venous outflow. Venous outflow impairment led to the formation of the collateral vessels on the right side of the neck. These changes caused muscle swelling, multiple small hemorrhages, and the formation of a new bleeding mass in the throat. ​​​​​​​

This case highlights the importance of taking into consideration a wide range of differential diagnoses for neck swelling during VV ECMO and the importance of drainage cannula positioning.

## Introduction

Veno-venous extracorporeal membrane oxygenation (VV ECMO) is a life-saving procedure used for patients with severe respiratory failure when conventional treatment fails [1]. Unfortunately, all forms of extracorporeal life support (ECLS) carry a risk of complications, which may occur during cannulation, ECMO therapy and decannulation. The most common complications include the following: bleeding from various sites, vessel damage, cardiac injury, arrhythmias, venous flow obstruction, thrombosis and thromboembolism, and ischemic issues [2,3]. Most of these can be life-threatening and require specific management, including surgical options. The goal in this situation is a fast diagnostic approach followed by the right tactical decision with further treatment.

Acute neck edema is a serious symptom for all critically ill patients, especially for those who are on ECMO [4]. This symptom can be a manifestation of a serious complication and should be diagnosed immediately and treated properly without delay. 

In this case report, we present a late cannulation-related complication accompanied by unilateral neck edema that occurred on the second day after ECMO cannulation.

Ultrasound-guided percutaneous peripheral cannulation is provided by ICU physicians at the bedside in our center.

## Case presentation

A 20-year-old woman with a normal BMI and acute myeloid leukemia in remission after bone marrow transplantation (day 120+) was admitted to the ICU with respiratory failure. Before admission, for the last two weeks, she had been an ambulatory patient and had been receiving cyclosporine as a specific treatment.

On day 0 in ICU, she required low-flow oxygen therapy. The next day, her hypoxemia worsened, and high-flow nasal oxygenation with prone positioning was initiated. Further, her respiratory condition continued to deteriorate, necessitating continuous positive airway pressure (CPAP), also with prone positioning. 

**Figure 1:**
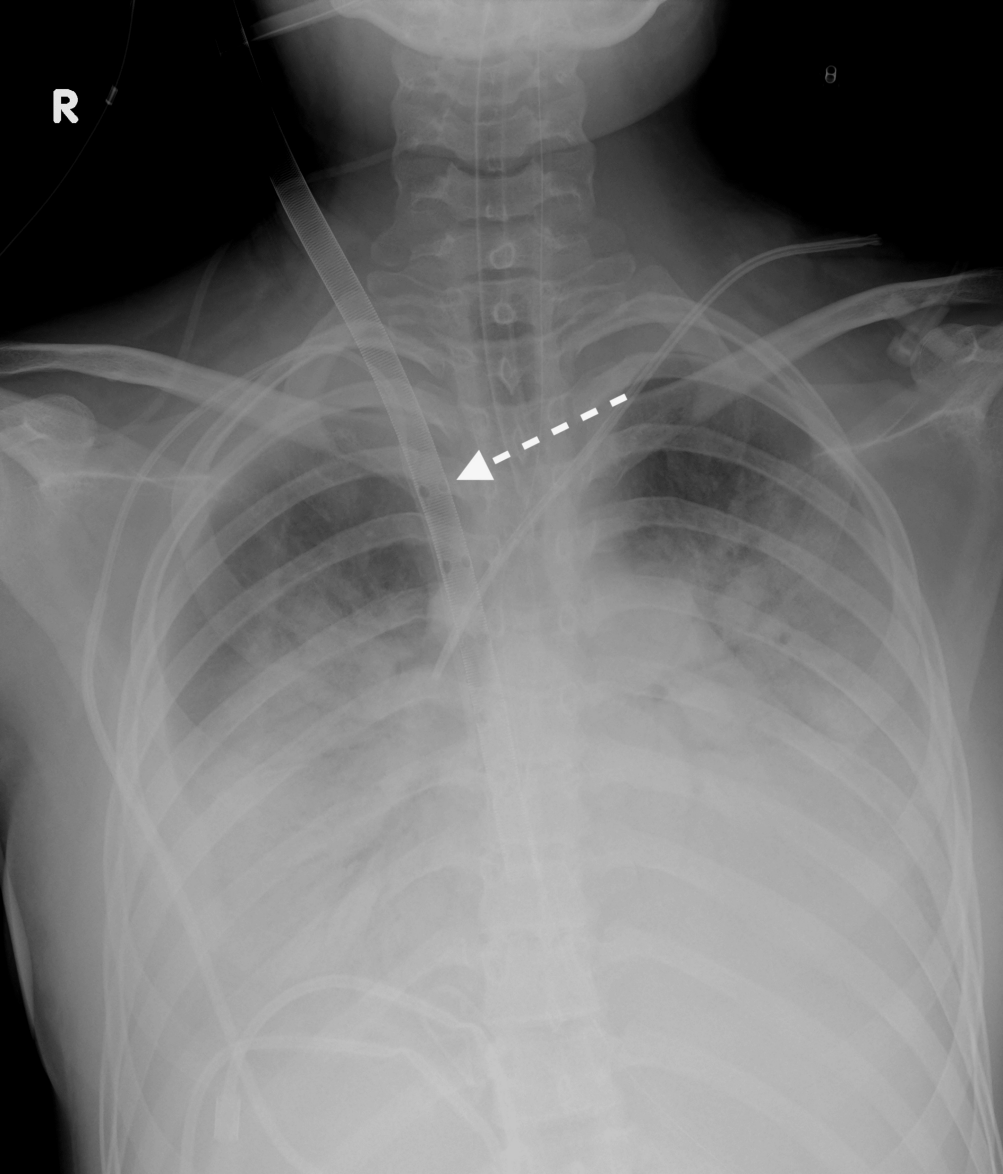
The X-ray after cannulas and central venous catheter (CVC) insertion The first side holes are positioned at the level of the clavicle.

On day 3, non-invasive ventilation methods had failed, and the patient was urgently intubated and invasive mechanical ventilation was provided. Rhinovirus and human herpesvirus 6 were detected by polymerase chain reaction (PCR) testing. Five hours after intubation, due to worsening hypoxemia and hypercarbia, VV ECMO was initiated. The right internal jugular vein (RIJV) (measuring 9.8x12.6 mm by ultrasound before cannulation) was cannulated with a 25Fr-38cm multistage (10cm) venous cannula for drainage. The previously placed 7Fr tunneled central venous catheter (CVC) on the same side was left in place due to the potential risk of hemorrhagic complications associated with catheter removal. The return was established through the right femoral vein using a 21Fr short arterial cannula. Cannulation was done without complication. The X-ray was used for positioning of drainage cannulas (Figure 1).

After cannulation and the initiation of ECMO, her clinical condition improved. However, malfunction of the tunneled CVC occurred, requiring the placement of a new CVC on the left side. A few hours later, renal replacement therapy (RRT) in continuous venovenous hemodiafiltration (CVVHDF) mode was initiated due to progressive azotemia and oliguria. The patient received a heparin bolus followed by continuous infusion to maintain an activated partial thromboplastin time (aPTT) of 60-70 seconds. ECMO functioned stably, with flows of 3.8-4.2 L/min at 2800-3000 RPM.

On day 4, the patient was extubated, spontaneously breathing with low-flow oxygen support via nasal cannula (0.5-1 L/min) while awake ECMO was provided. Oxygen saturation (SpO_2_) remained at 93-94%. A cough with minor hemoptysis developed a couple of hours after extubation, prompting an ENT evaluation, which revealed small blood clots in the posterior pharynx. Clots were removed with no further bleeding. 

**Figure 2:**
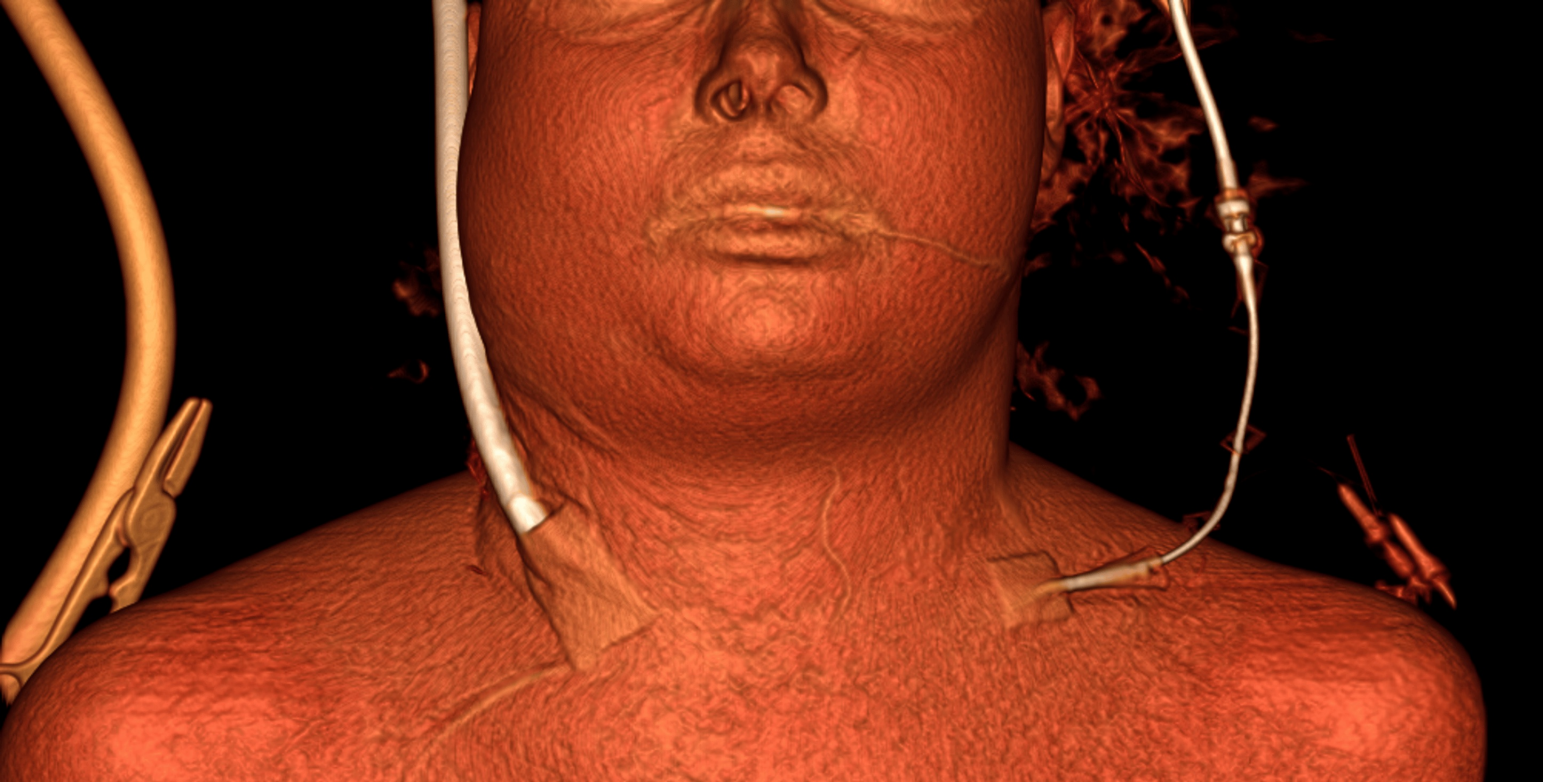
The 3D computed tomography model of the patient Unilateral right-sided neck edema is shown.

On the morning of day 5 (day 2 on ECMO), a large right-sided neck edema occurred (Figure 2).

**Figure 3:**
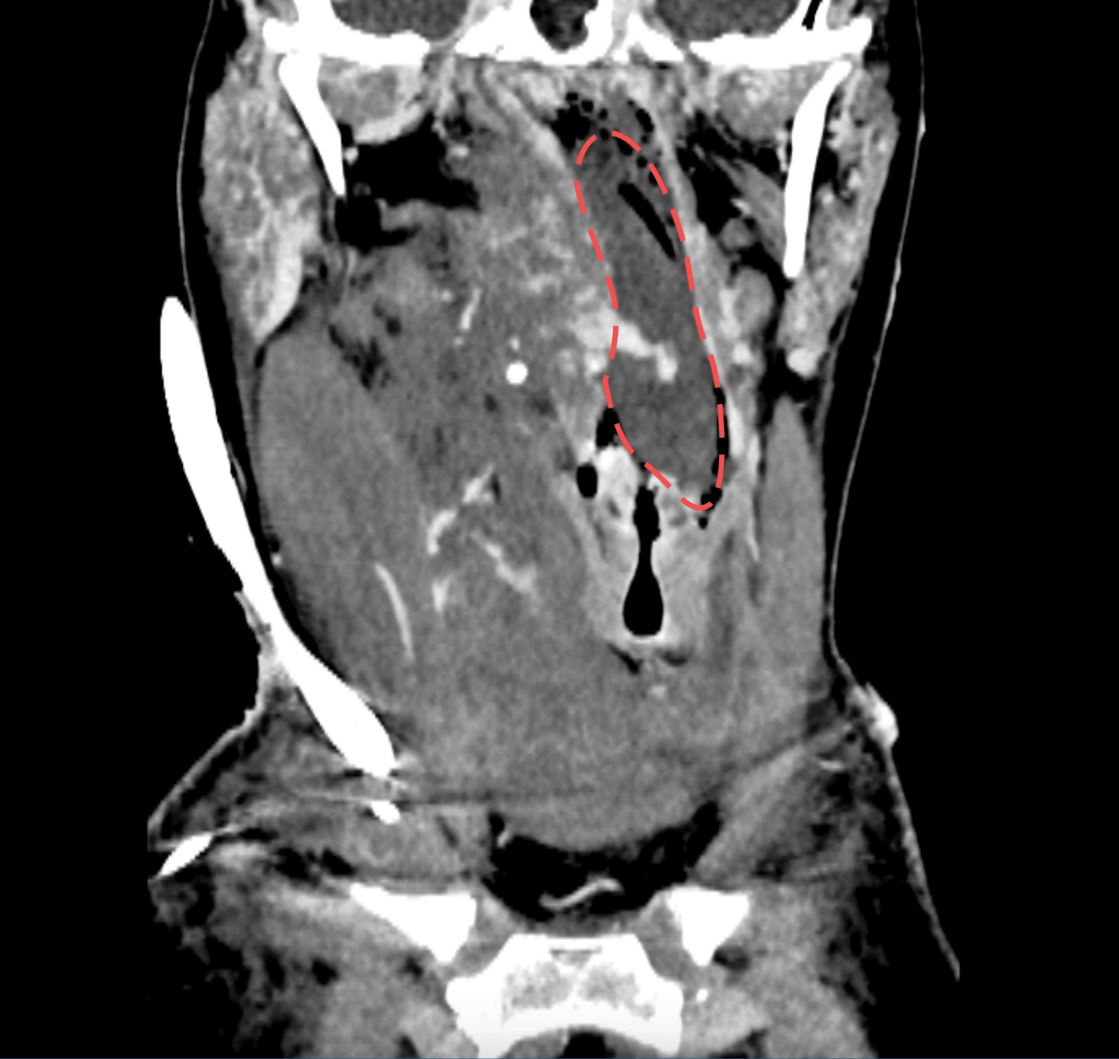
The coronal CT scan Unclear mass in throat marked with red lines.

There was no visible bleeding at the cannulation site, but the cough became more productive and hemorrhagic. Heparin infusion was stopped. ENT examination revealed bleeding in the throat and an unclear mass on the soft palate. A CT scan with contrast revealed muscle swelling accompanied by numerous small hemorrhages in the right side of the neck, resulting in significant tracheal deviation to the left. However, no active severe contrast extravasation was observed. The previously mentioned unclear mass resembled a hematoma, located near the entrance to the trachea (Figure 3, Video 1).

**Video 1:** The CT scans right after complication The CT scan with contrast (early phase), at the top; the CT scan with contrast (late phase), at the bottom.

**Figure 4:**
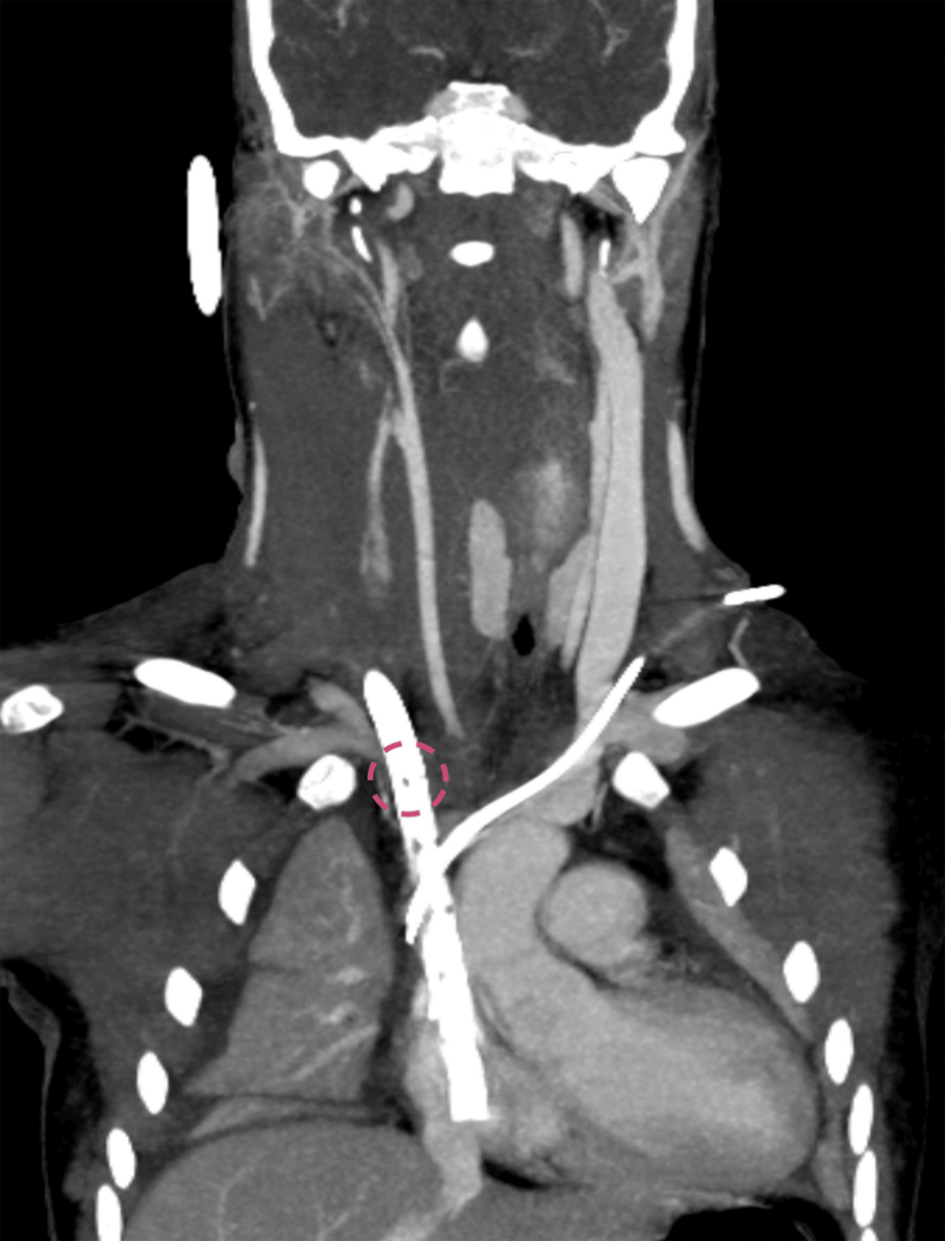
The coronal CT scan The first side holes are positioned below the junction of the jugular vein and the subclavian vein.

Further investigation identified partial thrombosis in the RIJV above the drainage cannula. Additionally, the first side hole of the drainage cannula was positioned below the junction of the RIJV and subclavian vein, which likely impaired venous outflow from the jugular vein (Figure 4).

**Figure 5:**
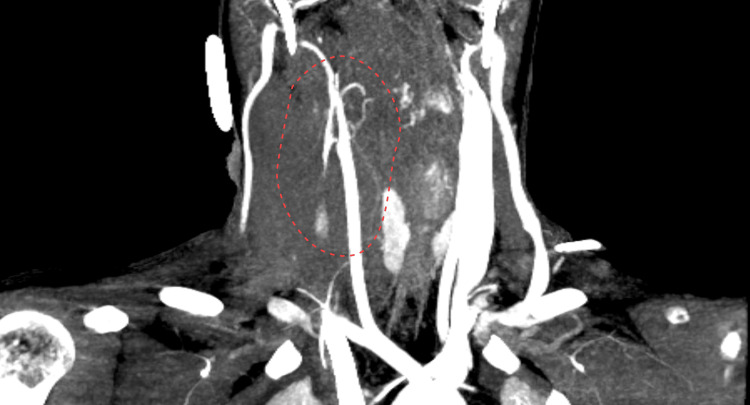
The coronal CT scan New collateral vessels in right side of the neck are partially shown.

New collateral vessels with signs of hyperemia developed in the right neck area at the level of the throat (Figure 5, Video 1).

**Figure 6:**
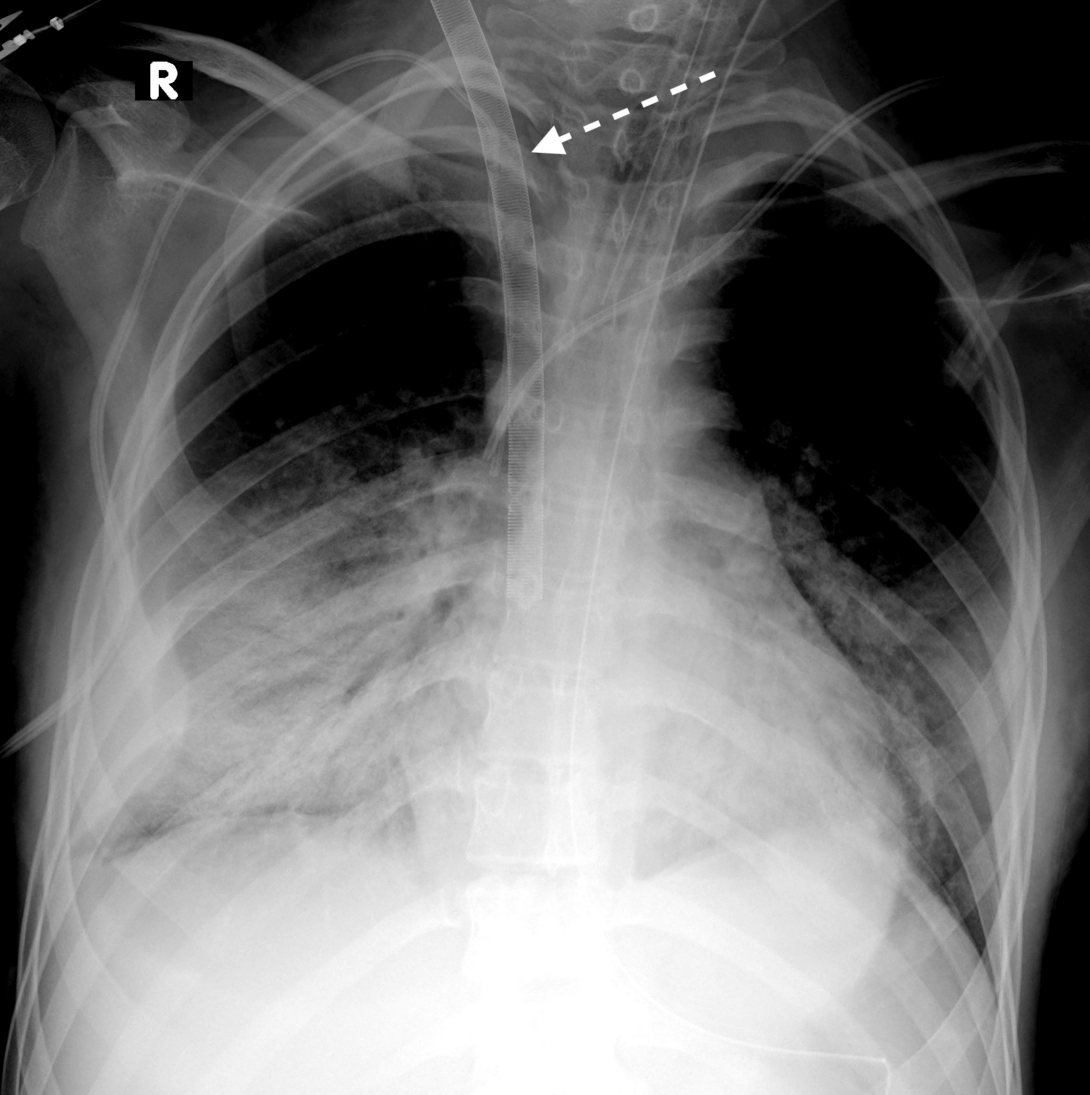
The X-ray after cannula retraction The first side holes are positioned above the clavicle.

The patient was re-intubated to secure the airway, and the drainage cannula was slightly retracted (Figure 6).

**Figure 7:**
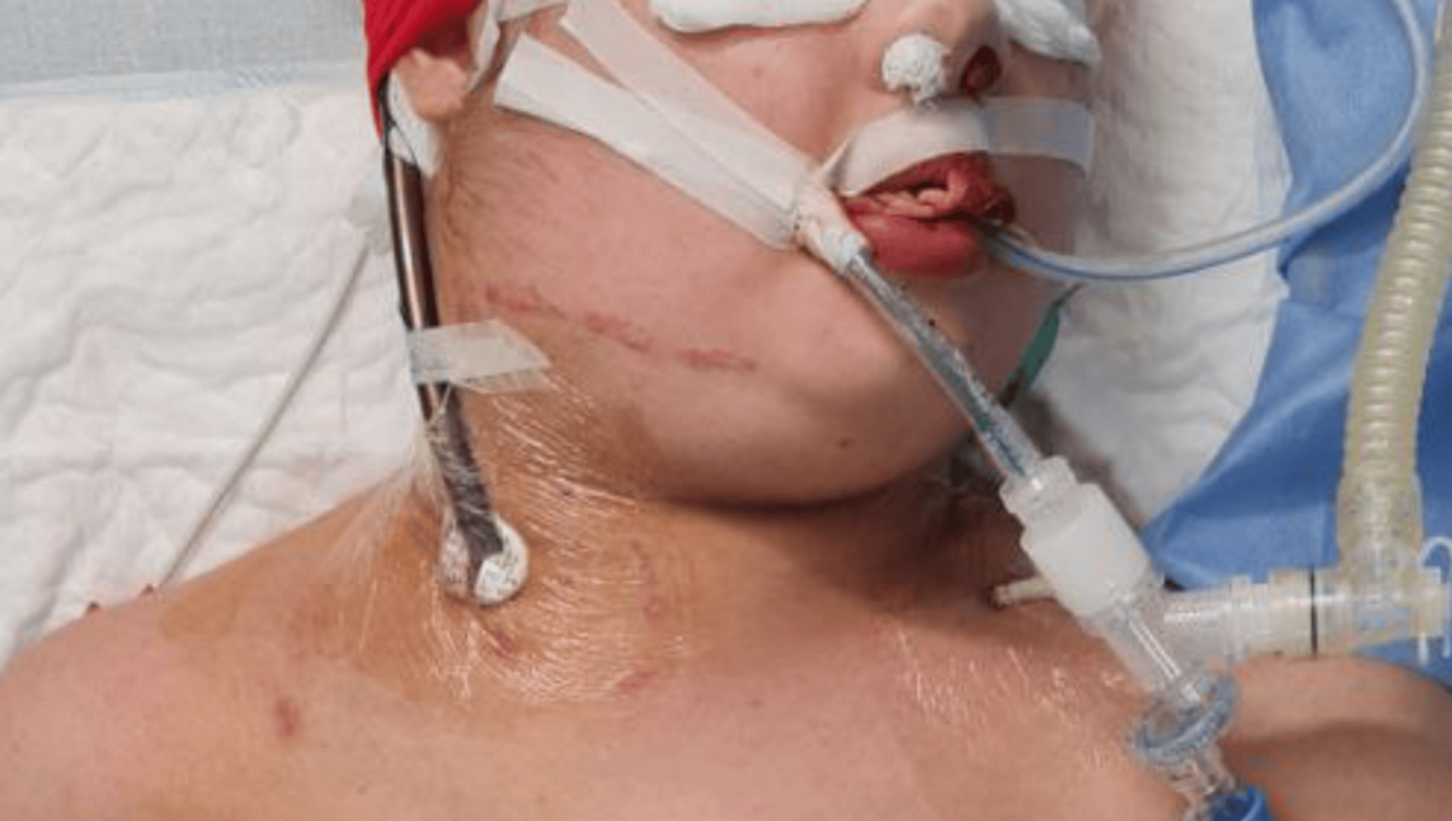
The picture of the patient after re-intubation and nasal-oral cavity packing Unilateral neck edema without external bleeding is shown.

Endoscopic examination showed no pulmonary or gastrointestinal bleeding. ENT performed nasal and oral cavity packing (Figure 7), and no-heparin ECMO was continued.

The patient received red blood cell and platelet transfusions to manage hemorrhagic syndrome caused by bleeding in the throat (Table 1).

**Table 1:** Laboratory Results and Transfusions During Extracorporeal Membrane Oxygenation (ECMO) Run Hb – haemoglobin; RBC – red blood cell; WBC – white blood cells; aPTT – activated partial thromboplastin time; INR – international normalised ratio.

	Day 0 (before ECMO cannulation)	Day 0 (after ECMO cannulation)	Day 1	Day 2 (complication)	Day 2 (12h later)	Day 3	Day 4	Day 4 (before decannulation)	Day 5
Hb (g/dl)	6.8	11.1	11.4	11.2	10.1	8.5	10.7	10.8	9.8
Platelets (×10^9^/l)	84	64	66	61	100	50	83	69	46
WBC (×10^9^/l)	8.94	6.84	7.23	9.32	13.4	6.8	8.25	8.77	7.24
aPTT	24.6	77.6	65.7	78.3	23.2	27.7	16.6	25	24.4
Fibrinogen (g/l)	5.55	5.04	5.2	4.38	3.96	3.61	3.18	3.07	2.35
INR	1.65	1.61	1.5	1.4	1.4	1.18	1.09	1.05	1.03
Therapy	RBC - 4 units; Platelets - 1 apheresis unit			RBC - 1 unit; Platelets - 1 apheresis unit; Heparin stopped		RBC - 1 unit; Platelets - 1 apheresis unit			Platelets - 1 apheresis unit

During days 6 and 7 (days 3-4 on ECMO), the neck swelling did not progress, and ECMO flows remained stable at 3.7-3.9 L/min with RPMs of 2500-2700.

On day 8 (day 5 on ECMO), the patient was successfully weaned from ECMO and decannulated without complications. A new CVC for RRT was inserted in the left femoral vein. The tunneled CVC resumed functioning after the drainage cannula was removed.

**Figure 8:**
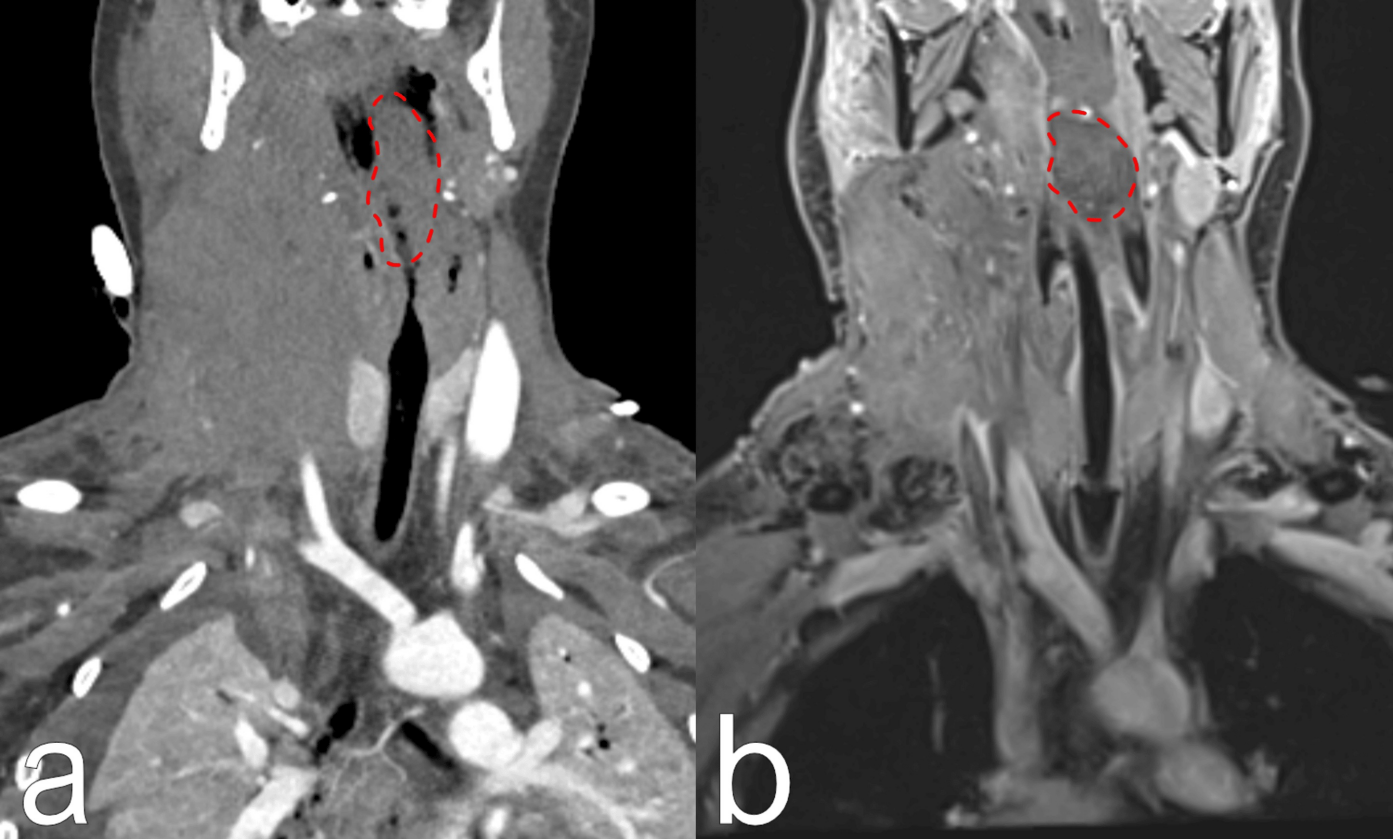
Patient's MRI and CT scans a) The CT scan on the day of complication; b) The MRI after decannulation from extracorporeal membrane oxygenation (ECMO) An unclear mass in the throat is marked with red lines.

On day 9, an MRI (Figure 8, Video 2B) revealed regression of the collateral vessels on the right side of the neck, normalization of the trachea’s position, reduced muscle swelling, and partial recanalization of the lower part RIJV. The previously mentioned unclear mass remained near the trachea entrance.

**Video 2:** Patient's MRI and CT scans a) The CT scan on the day of complication; b) The MRI after decannulation from extracorporeal membrane oxygenation (ECMO)

**Figure 9:**
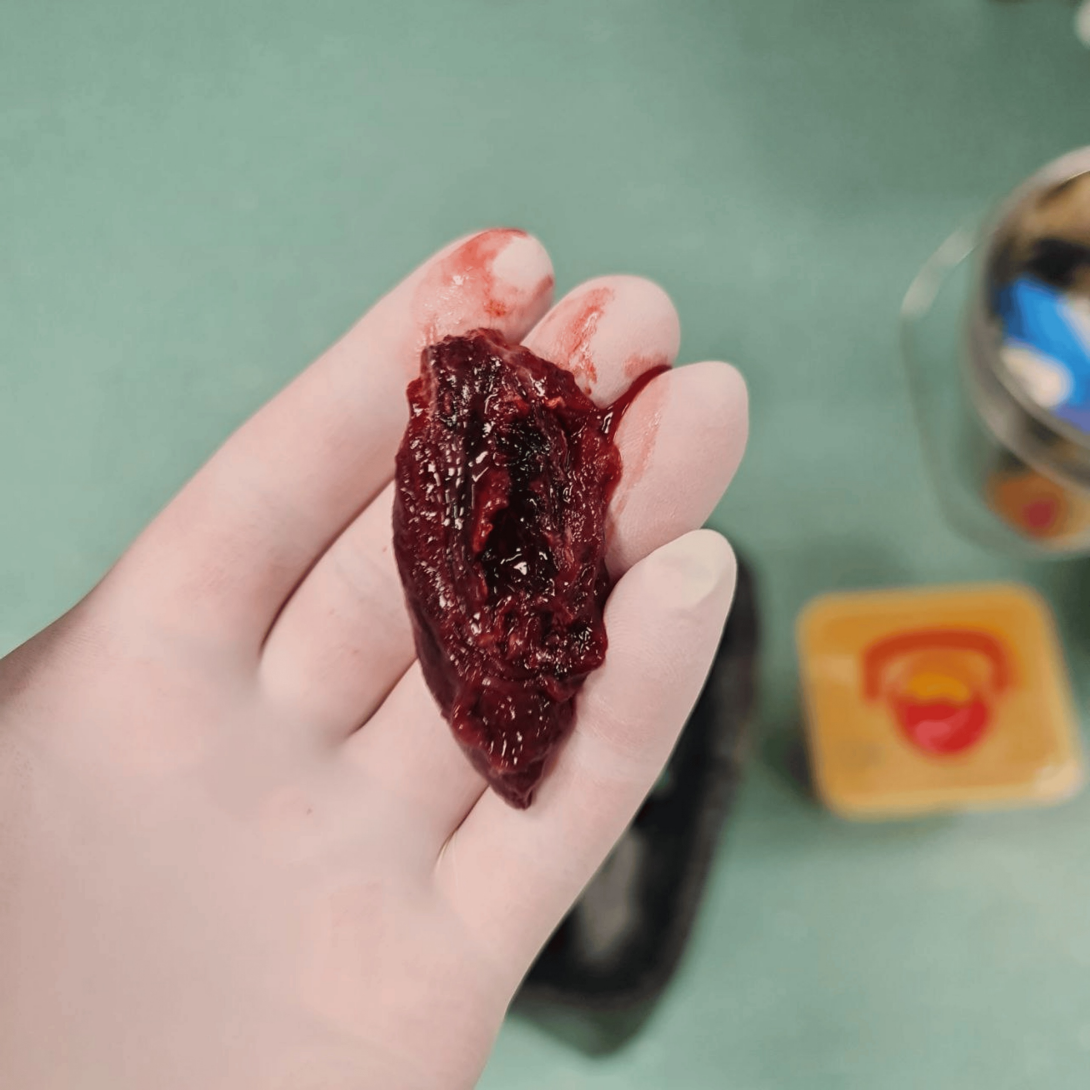
Removed mass from the throat before extubation.

On day 10, the ENT team removed the mass (Figure 9) without further bleeding and the patient was extubated.

**Figure 10:**
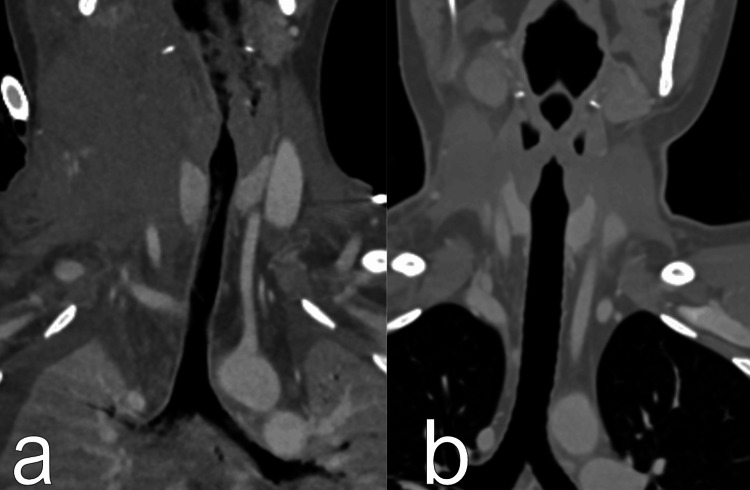
Patient's CT scans a) The CT scan on the day of complication; b) The CT scans one month later

No invasive procedures were performed on the neck or vessels regarding this complication. On day 11, the patient was transferred to the medical ward with ongoing anticoagulation therapy (prophylactic low-molecular-weight heparin). She was subsequently discharged to outpatient treatment. One month post-discharge, there were no issues with the throat or neck, and CT scans showed improvement in superior vena cava (SVC) patency (Figure 10, Video 3A, 3B). Histology of the mass revealed necrotic tissue with blood-filled cavities, muscle tissue, and bacterial colonies.

**Video 3:** The CT scans one month later a) the axial view; b) the coronal view

## Discussion

Following this case, a multidisciplinary review was conducted, involving specialists from the ICU, radiology, ENT, and oncology. It was concluded that the complication arose due to partial obstruction of venous outflow caused by both the drainage cannula and the tunneled CVC placement. This impaired outflow led to collateral vessel formation with hyperemia on the right side of the neck, causing muscle swelling, multiple small hemorrhages, and the formation of a new bleeding mass in the throat. Additionally, all hemorrhagic complications that happened, were exacerbated by the hemato-oncological condition of the patient.

The most frequent complications related to venous cannulation are thrombosis and bleeding, including bleeding from the insertion site [3,5]. Other bleeding sites, such as the GI tract, ear-nose-throat region, tracheostomy site, and intracranial bleeding, could be related to the patient's premorbid state, ICU treatment, and ECMO therapy in general [3]. The frequency of these complications can vary across different centers [6].

Additionally, other severe complications with neck edema following ECMO initiation, such as pneumomediastinum with pneumothorax and subcutaneous emphysema, which were common for COVID-19 patients, must also be considered [7].

Neck edema during VV ECMO is a dangerous clinical symptom, and first of all active bleeding in the neck space and subcutaneous emphysema due to mediastinal organ damage must be excluded. 

Additionally, SVC obstruction syndrome, although rare in the adult population [8], and various pathological processes in the thyroid gland [3], should be considered.

Most likely, our case involves a combined type of complication, including late unilateral neck edema and subsequent hemorrhage from the throat related to partial obstruction of the venous system and to unusual formation of collateral vessels. 

## Conclusions

We present a rare case of delayed acute unilateral neck edema that developed during VV ECMO. This case underscores the importance of considering a wide range of differential diagnoses for neck swelling and exploring other potential causes, as they may significantly affect both VV ECMO management and the patient's condition. 

We would also like to emphasize the importance of venous outflow monitoring while draining from the SVC system, due to the unobvious possibility of developing an outflow obstruction and venous congestion, which could lead to secondary hemorrhagic complications.
